# Knowledge and Attitudes of Health Care Providers Toward Transgender Patients Within a Rural Tertiary Care Center

**DOI:** 10.1089/trgh.2018.0050

**Published:** 2019-02-04

**Authors:** Shon P. Rowan, Christa L. Lilly, Robert E. Shapiro, Kacie M. Kidd, Rebecca M. Elmo, Robin A. Altobello, Manuel C. Vallejo

**Affiliations:** ^1^ Department of Obstetrics and Gynecology, School of Medicine, West Virginia University, Morgantown, West Virginia.; ^2^ Department of Biostatistics, School of Public Health, West Virginia University, Morgantown, West Virginia.; ^3^ Department of Pediatrics, School of Medicine, West Virginia University, Morgantown, West Virginia.; ^4^ Department of Medical Education, School of Medicine, West Virginia University, Morgantown, West Virginia.; ^5^ Department of Occupational Medicine, School of Public Health, West Virginia University, Morgantown, West Virginia.; ^6^ Department of Medical Education, Anesthesiology, Obstetrics and Gynecology, School of Medicine, West Virginia University, Morgantown, West Virginia.

**Keywords:** attitudes, health providers, LGTB, survey, transgender

## Abstract

**Purpose:** Members of the transgender community face significant health disparities within our society, especially within the state of West Virginia, which is primarily rural. We sought to examine and compare existing attitudes and knowledge of resident and faculty physician medical professionals at our institution about treating transgender individuals within a rural tertiary care center.

**Methods:** The Medical Practitioner Attitudes Towards Transgender Patients (MP-ATTS) survey and the Medical Practitioner Beliefs and Knowledge about Treating Transgender Patients (MP-BKTTP) survey were sent to all faculty and resident physicians at West Virginia University Hospitals. Demographics included information about gender, rurality of hometown, race, and description of medical practitioner status (i.e., years out of residency, residency status).

**Findings:** In general, there were positive attitudes and reception of the survey among residents and faculty physicians. 76.45% of providers assumed that their patients were not transgender. More than 40% of respondents believed that they would need further education about transgender patients to provide appropriate health care. Male health care providers had significantly higher negative perceptions of the transgender community (*N*=85, M=4.46, standard deviation [SD]=0.55, *p*<0.0001) and perceived fewer barriers due to personnel (*N*=80, M=3.24, SD=0.96, *p*<0.0001).

**Conclusion:** A clear need exists for increased training in transgender health care among physicians taking care of this patient population. A gender divide exists among health care providers within West Virginia over attitudes regarding the transgender community. Further studies are needed to fully understand the health care needs and barriers of the transgender population.

## Introduction

Members of the transgender community face significant health disparities within our society. In 2011, the Institute of Medicine (IOM) issued a report which states that not only do the transgender population experience health care disparities when compared with their cisgender peers, the type and extent of these disparities vary across age and specific orientation.^1^ Consequently, the transgender community are more likely to avoid or delay seeking health care compared with heterosexuals.^2^ They are also less likely to have health insurance coverage than their cisgender counterparts, despite the passage of the Affordable Care Act.^3^ This has ultimately resulted in treatment delays and overall poorer health outcomes for this community.^4^

Perceived discrimination, personal bias, and negative attitudes from health care professionals interfere with health care delivery to the transgender community.^5^ Many members of this community choose not to disclose their sexual orientation or gender identity to health care providers given the frequency of experiencing negative attitudes through both overt and covert forms of discrimination.^6^ Many transgender individuals also report intense anxiety related to disclosure of sexual identity to health care providers, fearing that such disclosure will make them vulnerable to mistreatment and denial of care.^7^

Geography is central to forming perceptions regarding the transgender community. Research focused on the influence of geography on the transgender culture shows that rural lifestyle can increase the likelihood of isolation and intolerance of the transgender community, whereas more urban areas are associated with an increased acceptance and willingness to “come out.”^8^ Little is known, however, about the role geography plays within the delivery of health care to this patient population.

The Health Disparities Working Group (HDWG) at West Virginia University (WVU) was established to foster multidisciplinary collaboration among researchers and practitioners across West Virginia. Most patients treated at West Virginia University Hospital are from rural West Virginia and have many of the inequities associated with that population.^9^ Our primary objective was to examine the existing attitudes and knowledge of medical professionals in the care and treatment of transgender individuals within a rural tertiary care center. As a secondary outcome, we compared results from two cohorts that provide medical services at WVU: residents and faculty physician providers.

## Methods

In 2016, an Internet survey was sent to all faculty and resident physicians at West Virginia University Hospitals. Local Investigative Review Board (IRB) approval was obtained for the survey (protocol no. 1708703263), and the survey was administered electronically via the Study, Observe, Learn, Engage (SOLE) university online medical education website.

The questions were designed after literature search for existing scales with established psychometric properties including a search strategy of the American Psychological Association database of abstracts of literature in the field of psychology (APA psycNET) within the disparity population of interest (Transgender) and then inclusion of medical terms, such as provider, medical, health, and hospital.

In addition to a demographic questionnaire, two surveys were used. The Medical Practitioner Attitudes Towards Transgender Patients (MP-ATTS) survey^10,11^ was used to measure general attitudes about transgender individuals. The MP-ATTS included 15 questions with 6 items reverse coded (higher scores=more positive attitudes). Just more than 50% of the variance was accounted for by a single component solution in a principal component analysis (PCA); all items loaded over 0.53 on the single factor solution. Internal reliability was excellent, α=0.92. The Medical Practitioner Beliefs and Knowledge about Treating Transgender Patients (MP-BKTTP) survey^12^ was used to assess general medical beliefs and knowledge about treating transgender patients. The MP-BKTTP included 13 questions with 6 items reverse coded (higher scores=more positive beliefs and knowledge). The scale did not load into a single factor PCA, and further factor analysis suggested two subscales from these items. For the two subscales, internal reliability was acceptable, α=0.94 (four items) for perceived barriers to care by personnel and α=0.71 (three items) for personal preference to treat. For consistency, both surveys utilized a 5-point Likert average ratings scale, where 1 is strongly disagree, 2 is disagree, 3 is neither agree or disagree, 4 is agree, and 5 is strongly agree. Demographics included information about gender, rurality of hometown, race, and description of medical practitioner status (i.e., years out of residency, residency status). Variables with multiple response options (such as gender and race) were dummy coded for analysis. A separate section for free text response was also used to give better insight to the general results of the standardized survey.

Only de-identified results were obtained. The questionnaire in its final form can be found in Appendix A1. All statistical analyses were conducted using SAS 9.4. Internal reliability for items within scales was checked using standardized Cronbach's alpha with complete data. PCA was conducted to assess the items appropriateness for scale use. Exploratory factor analysis, using maximum likelihood estimation with promax rotation, was conducted as a follow-up to the PCA. Items were averaged together if participants greater or equal to 75% response across items for scales and subscales. Descriptive statistics included frequency and valid percent for categorical items and demographics and means and standard deviations for scale scores. Pearson correlations were used to check the association between the scales. Analysis of variance and independent sample *t*-tests were conducted between demographics and the scale scores, alpha set to 0.05.

## Results

Two samples were included in the survey; first, all residents (*N*=424) were e-mailed, with *N*=79 responding (for a total response rate of 18.6%). Next, all WVU faculty physicians (*N*=648) were e-mailed the survey, with *N*=129 responding (for a response rate of 19.9%). All data were combined for a total sample of 208 responses.

Item by item responses and demographics are given in Table 1, including number of participants endorsing the category with the valid percentage reported. Notably, strong positive patterns of endorsement were seen for items such as *Transgender patients deserve the same level of quality care from medical institutions as cisgender patients* (84% strongly agree), *I would prefer not to treat transgender patients* (69% strongly disagree), and *I am willing to treat transgender patients within my scope of practice* (69% strongly agree). More varied patterns of agreement were seen on all other items. It is also noteworthy that 76.45% of providers assumed that their patients were not transgender and more than 40% responded that they would need further education about transgender patients to provide appropriate medical care for them.

**Table 1.** Frequency and Percentage Endorsing of All Survey Items and Demographics

Scale/subscale	Item	Reverse coded					
MP-BKTTP (beliefs and knowledge)			Strongly disagree	Disagree	Neither agree nor disagree	Agree	Strongly agree
	Q1. As a medical provider, it is important for me to know about my patients' gender identity.		14 (6.8%)	13 (6.31%)	34 (16.5%)	84 (40.78%)	61 (29.61%)
	Q2. When I first meet someone, I assume they are cisgender (nontransgender).	Y	8 (3.85%)	11 (5.29%)	30 (14.42%)	111 (53.37%)	48 (23.08%)
	Q3. Transgender patients deserve the same level of quality care from medical institutions as cisgender patients.		6 (2.88%)	2 (0.96%)	2 (0.96%)	24 (11.54%)	174 (83.65%)
Perceived barriers to care by personnel	Q4. At WVU Medicine, I am aware of physicians or advanced practice providers who exhibit attitudes or beliefs about the transgender population that I feel are barriers to care.	Y	41 (20.71%)	67 (33.84%)	45 (22.73%)	33 (16.67%)	12 (6.06%)
Perceived barriers to care by personnel	Q5. At WVU Medicine, I am aware of front desk staff who exhibit attitudes or beliefs about the transgender population that I feel are barriers to care.	Y	37 (19.27%)	73 (38.02%)	57 (29.69%)	20 (10.42%)	5 (2.6%)
Perceived barriers to care by personnel	Q6. At WVU Medicine, I am aware of nursing staff who exhibit attitudes or beliefs about the transgender population that I feel are barriers to care.	Y	44 (22.45%)	59 (30.1%)	56 (28.57%)	28 (14.29%)	9 (4.59%)
Perceived barriers to care by personnel	Q7. At WVU Medicine, I am aware of facilities staff who exhibit attitudes or beliefs about the transgender population that I feel are barriers to care.	Y	41 (22.53%)	60 (32.97%)	62 (34.07%)	15 (8.24%)	4 (2.2%)
	Q8. Have you ever been involved in the treatment of a transgender patient?		17 (8.42%)	25 (12.38%)	15 (7.43%)	77 (38.12%)	68 (33.66%)
	Q9. I would need to be better educated about transgender patients to provide appropriate medical care for them.		35 (17.07%)	53 (25.85%)	33 (16.1%)	71 (34.63%)	13 (6.34%)
	Q10. Transgender patients have unique health risks and needs.		9 (4.33%)	14 (6.73%)	18 (8.65%)	110 (52.88%)	57 (27.4%)
Personal preference to treat	Q11. I am willing to treat transgender patients within my scope of practice.		1 (0.48%)	2 (0.96%)	6 (2.88%)	56 (26.92%)	143 (68.75%)
Personal preference to treat	Q12. I would prefer not to treat transgender patients.	Y	139 (69.15%)	49 (24.38%)	9 (4.48%)	2 (1%)	2 (1%)
Personal preference to treat	Q13. I am comfortable treating transgender patients.		3 (1.46%)	5 (2.44%)	34 (16.59%)	85 (41.46%)	78 (38.05%)

MP-ATTS (general attitudes)			Strongly Disagree	Disagree	Neither Agree nor Disagree	Agree	Strongly Agree
	Q14. It would be beneficial to society to recognize the state of being transgender as natural.		14 (6.97%)	23 (11.44%)	45 (22.39%)	62 (30.85%)	57 (28.36%)
	Q15. Transgender individuals should not be allowed to work with children.	Y	112 (54.63%)	59 (28.78%)	20 (9.76%)	6 (2.93%)	8 (3.9%)
	Q16. Transgender individuals are immoral.	Y	143 (70.79%)	41 (20.3%)	11 (5.45%)	5 (2.48%)	2 (0.99%)
	Q17. Transgender individuals are viable and contributing members of our society.		4 (1.94%)	3 (1.46%)	14 (6.8%)	75 (36.41%)	110 (53.4%)
	Q18. Transgender individuals endanger the institution of the family.	Y	125 (61.27%)	45 (22.06%)	17 (8.33%)	14 (6.86%)	3 (1.47%)
	Q19. Transgender individuals should be accepted completely into our society.		6 (2.96%)	4 (1.97%)	24 (11.82%)	68 (33.5%)	101 (49.75%)
	Q20. Transgender individuals should be barred from the teaching profession.	Y	141 (68.12%)	51 (24.64%)	12 (5.8%)	2 (0.97%)	1 (0.48%)
	Q21. There should be no restriction of rights for transgender individuals.		5 (2.43%)	10 (4.85%)	10 (4.85%)	57 (27.67%)	124 (60.19%)
	Q22. I avoid transgender individuals whenever possible.	Y	127 (61.65%)	57 (27.67%)	19 (9.22%)	2 (0.97%)	1 (0.49%)
	Q23. I would feel comfortable working closely with a transgender individual.		2 (0.98%)	8 (3.9%)	20 (9.76%)	81 (39.51%)	94 (45.85%)
	Q24. I would feel comfortable if I learned that my neighbor was a transgender individual.		3 (1.45%)	5 (2.42%)	14 (6.76%)	76 (36.71%)	109 (52.66%)
	Q25. Transgender individuals should be allowed to dress to reflect their gender identity in public.		3 (1.49%)	3 (1.49%)	22 (10.89%)	70 (34.65%)	104 (51.49%)
	Q26. I would feel comfortable if I learned that my best friend was a transgender individual.		5 (2.46%)	11 (5.42%)	26 (12.81%)	76 (37.44%)	85 (41.87%)
	Q27. I would feel uncomfortable if a close family member became romantically involved with a transgender individual.	Y	51 (25.25%)	56 (27.72%)	38 (18.81%)	34 (16.83%)	23 (11.39%)
	Q28. I would feel comfortable if a close family member was a transgender individual.		11 (5.45%)	21 (10.4%)	31 (15.35%)	73 (36.14%)	66 (32.67%)

Demographics		*N*	Valid %
	Faculty	129	—
	Missing data	—	—
	Prefer not to answer	2	1.54
	Less than 5 years out of residency	27	20.77
	5–10 Years out of residency	25	19.23
	10–20 Years out of residency	31	23.85
	20 or more years out from residency	45	34.62
	Residents	79	—
	Missing data	2	—
	Prefer not to answer	4	5.19
	PGY-1	27	34.18
	PGY-2	15	19.48
	PGY-3	14	18.18
	PGY-4 or higher	17	22.08
	Hometown		
	Prefer not to answer	3	1.46
	Large metro (city of 1 million population or more)	25	12.20
	Mid-sized metro (city of 250,000–1 million population)	19	9.27
	Small-sized metro (city of fewer than 250,000 population)	21	10.24
	Urban (20,000–250,000 in the county)	64	31.22
	Small urban (i.e., town) (2500–20,000 in the county)	48	23.41
	Rural (less than 2500 in county)	25	12.20
	Gender (more than one option can be selected)		
	Male	121	58.45
	Female	83	40.10
	All other categories	3	1.45
	Ethnicity		
	Prefer not to answer	13	6.31
	Hispanic or Latino	5	2.43
	Not Hispanic or Latino	188	91.26
	Race (more than one option can be selected)		
	Missing	13	—
	Asian	16	8.21
	Black or African American	4	2.05
	White (Europe/Middle East/North Africa)	169	86.67
	Other	32	16.41
	Faculty only (more than one can be selected)	—	—
	Know transgender individuals?	—	—
	Prefer not to answer	4	2.50
	Workplace leadership/supervisor	3	1.88
	Colleague/co-worker 22 (13.75%)	22	13.75
	Family/friend	32	20.00
	Patient	57	35.62
	I do not know any transgender individuals	42	26.25

MP-ATTS, Medical Practitioner Attitudes Towards Transgender Patients; MP-BKTTP, Medical Practitioner Beliefs and Knowledge about Treating Transgender Patients; WVU, West Virginia University.

Relationships between scale scores with demographics and specific items were explored next, as shown in Table 2. Notably, only two significant differences were found among the tested differences. Males had lower average general attitudes on the MP-ATTS (*N*=119, M=4.05, standard deviation [SD]=0.70) than females or other genders (*N*=85, M=4.46, SD=0.55), *t*=4.50, *p*<0.0001. Males also perceived fewer barriers due to personnel (*N*=111, M=3.79, SD=0.94) than females or other genders (*N*=80, M=3.24, SD=0.96), *t*=−3.97, *p*<0.0001.

**Table 2.** Analysis of Variance Results on Scales and Subscales by Selected Demographic Characteristics

Outcome	Group	*N*	*F*-value	*p*	*R*^2^
MP-ATTS					
	Form (faculty vs. resident)	204	0.58	0.48	0.003
	Male	203	20.24	<0.0001	0.091
	Hometown	201	186	0.09	0.054
	Race (white vs. Other)	192	0.04	0.83	0.000
Perceived barriers by personnel					
	Form (faculty vs. resident)	191	3.16	0.08	0.016
	Male	190	15.76	<0.0001	0.077
	Hometown	188	1.52	0.17	0.048
	Race (white vs. Other)	180	1.27	0.26	0.007
Personal preference to treat					
	Form (faculty vs. resident)	207	0.35	0.55	0.002
	Male	206	0.56	0.46	0.003
	Hometown	204	1.95	0.07	0.056
	Race (white vs. Other)	195	1.38	0.24	0.007

Finally, we wanted to look at the association between general attitudes on the MP-ATTS and specific medical practices on the MP-BKTTP. Expected significant associations were found, including a Pearson correlation *r*=−0.17, *p*=0.02, between general attitudes and perceived barriers to care by personnel (*N*=190), indicating that more positive general attitudes perceived more barriers. A strong significant correlation was also found between general attitudes and personal preference to treat, *r*=0.61, *p*<0.0001 (*N*=250).

Text responses in the notes section also gave unique insight into these general results. In response to the question “How could WVU improve the care we provide to transgender patients?” Responses were given by 49 participants (23.6% response). Participant responses were largely positive and centered on education, such as through grand rounds, curriculum for students, staff, and faculty education trainings. Other positive responses included access initiatives, such as changing the autopopulated gender responses in the electronic medical system or increasing nongender bathrooms. Some responses denoted neutral responses such as no changes, no idea, not an issue, or there is no difference in care due to transgender status.

## Discussion

In general, we had very positive attitudes and reception of the survey among WVU residents and faculty physicians. As expected, general attitudes were related to aspects of medical care and knowledge. This is important because data are limited on attitudes regarding transgender health care, especially within a rural population.^13^

An interesting finding in our study revealed that 76.45% of providers assumed that their patients are not transgender. This raises some concerns about health care delivery to the transgender community within West Virginia. Members of the transgender community experience higher rates of smoking, alcohol, and substance abuse and are at higher risk for mental health issues, sexually transmitted diseases, and increased incidence of some cancers.^14^ These results suggest that intake evaluations for all patients should be gender inclusive and history taking by the clinician be trans-sensitive.

More than 40% of our survey respondents believed that they needed to be better educated about transgender health issues to provide appropriate medical care for the transgender community. This points further to the need for increased training in transgender health.

In our study, male health care providers had significantly higher negative perceptions of the transgender community and perceived fewer barriers due to personnel. This seems to correlate with other published literature on this topic, not unique to health care providers. Norton and Herek showed that attitudes toward the transgender population were more negative among heterosexual men than women.^15^ In their regression analysis, sexual prejudice accounted for much of the variance.

Size of health care provider's hometown did not have significant influence over the propensity to treat transgender patients. Perhaps, an increasingly geographical diversity and visibility of transgender individuals throughout rural, urban, and metropolitan communities alike played a role in this study finding.

Although the authors know of no other research that focuses on perceptions of health care providers on a transgender patient population within a rural tertiary care setting, selection bias and generalizability of the sample to a broader setting are limitations of the study. The attitudes and beliefs of health care providers of a more urban socioeconomic status could differ considerably. Also, advanced practice clinicians, who follow different training tracks, could also have differing attitudes.

Another limitation of this study is the lack of existing validated appropriate measures. We used established scales where possible for this research; however, in some measures, we needed to change some of the words to suit our research questions or to update language. For example, certain items were dropped, and other items were added to complete certain subconcepts within the scale. Other terms were changed or adapted; for example, changing the term in some items from “transgendered” to “transgender” to denote it is not possible to be “gendered,” and changing the word heterosexual to cisgender to more accurately reflect gender rather than sexuality. A systematic approach was taken to basic content validation, including face validation of the items and consultation with experts in the field, and good internal reliability estimates and basic validation of items using factor analysis were obtained. However, future research should further assess the validity of these adapted scales.

## Conclusion

Health care systems need to be mindful of the availability, accessibility, acceptability, and equity of their services. Even where clinical services for transgender patients are available, quality of care may vary depending on the educational training and attitudes of the health care providers toward this patient population. Further studies that include larger numbers of individuals across broader geographical areas are needed to fully understand the health care needs and barriers of the transgender population.

## Abbreviations Used

MP-ATTS: Medical Practitioner Attitudes Towards Transgender Patients
MP-BKTTP: Medical Practitioner Beliefs and Knowledge about Treating Transgender Patients
PCA: principal component analysis
SD: standard deviation
WVU: West Virginia University

## Appendix A1. Printable Survey—Transgender Health Care Disparities Assessment

### Experiences at West Virginia University Medicine

Instructions: The first 13 questions are designed to measure the way you feel about your experiences with transgender individuals in the medical community at West Virginia University (WVU) Medicine. Read each item carefully and choose the option that most accurately reflects your feeling: 1=Strongly disagree, 2=Disagree, 3=Neither agree nor disagree, 4=Agree, 5=Strongly agree, or N/A=Prefer not to answer. This is not a test. There is no right or wrong answer to these questions.

1. As a medical provider, it is important for me to know about my patients' gender identity. (N/A=Prefer not to answer)
  N/A
  1: Strongly disagree
  2: Disagree
  3: Neither agree nor disagree
  4: Agree
  5: Strongly agree
2. When I first meet someone, I assume they are cisgender (nontransgender). (N/A=Prefer not to answer)
  N/A
  1: Strongly disagree
  2: Disagree
  3: Neither agree nor disagree
  4: Agree
  5: Strongly agree
3. Transgender patients deserve the same level of quality care from medical institutions as cisgender patients. (N/A=Prefer not to answer)
  N/A
  1: Strongly disagree
  2: Disagree
  3: Neither agree nor disagree
  4: Agree
  5: Strongly agree
4. At WVU Medicine, have you witnessed a physician or advanced practice provider exhibit attitudes or beliefs about the transgender population that you feel are barriers to care? (N/A=Prefer not to answer)
  N/A
  1: Strongly disagree
  2: Disagree
  3: Neither agree nor disagree
  4: Agree
  5: Strongly agree
5. At WVU Medicine, have you witnessed the front desk staff exhibiting attitudes or beliefs about the transgender population that you feel are barriers to care? (N/A=Prefer not to answer)
  N/A
  1: Strongly disagree
  2: Disagree
  3: Neither agree nor disagree
  4: Agree
  5: Strongly agree
6. At WVU Medicine, have you witnessed the nursing staff exhibiting attitudes or beliefs about the transgender population that you feel are barriers to care? (N/A=Prefer not to answer)
  N/A
  1: Strongly disagree
  2: Disagree
  3: Neither agree nor disagree
  4: Agree
  5: Strongly agree
7. At WVU Medicine, have you witnessed the facilities staff exhibiting attitudes or beliefs about the transgender population that you feel are barriers to care? (N/A=Prefer not to answer)
  N/A
  1: Strongly disagree
  2: Disagree
  3: Neither agree nor disagree
  4: Agree
  5: Strongly agree
8. Have you ever been involved in the treatment of a transgender patient? (N/A=Prefer not to answer)
  N/A
  1: Strongly disagree
  2: Disagree
  3: Neither agree nor disagree
  4: Agree
  5: Strongly agree
9. I would need to be better educated about transgender patients to provide appropriate medical care. (N/A=Prefer not to answer)
  N/A
  1: Strongly disagree
  2: Disagree
  3: Neither agree nor disagree
  4: Agree
  5: Strongly agree
10. Transgender patients have unique health risks and needs. (N/A=Prefer not to answer)
  N/A
  1: Strongly disagree
  2: Disagree
  3: Neither agree nor disagree
  4: Agree
  5: Strongly agree
11. I am willing to treat transgender patients within my scope of practice. (N/A=Prefer not to answer)
  N/A
  1: Strongly disagree
  2: Disagree
  3: Neither agree nor disagree
  4: Agree
  5: Strongly agree
12. I would prefer not to treat transgender patients. (N/A=Prefer not to answer)
  N/A
  1: Strongly disagree
  2: Disagree
  3: Neither agree nor disagree
  4: Agree
  5: Strongly agree
13. I am comfortable treating transgender patients. (N/A=Prefer not to answer)
  N/A
  1: Strongly disagree
  2: Disagree
  3: Neither agree nor disagree
  4: Agree
  5: Strongly agree

### Feelings About Working and Associating

Instructions: The following questions are designed to measure more generally the way you feel about working or associating with transgender individuals. Read each item carefully and choose the answer that most accurately reflects your feeling: 1=Strongly disagree, 2=Disagree, 3=Neither agree nor disagree, 4=Agree, 5=Strongly agree, or N/A=Prefer not to answer. This is not a test. There is no right or wrong answer to these questions.

14. It would be beneficial to society to recognize the state of being transgender as natural. (N/A=Prefer not to answer)
  N/A
  1: Strongly disagree
  2: Disagree
  3: Neither agree nor disagree
  4: Agree
  5: Strongly agree
15. Transgender individuals should not be allowed to work with children. (N/A=Prefer not to answer)
  N/A
  1: Strongly disagree
  2: Disagree
  3: Neither agree nor disagree
  4: Agree
  5: Strongly agree
16. Transgender individuals are immoral. (N/A=Prefer not to answer)
  N/A
  1: Strongly disagree
  2: Disagree
  3: Neither agree nor disagree
  4: Agree
  5: Strongly agree
17. Transgender individuals are viable and contributing members of our society. (N/A=Prefer not to answer)
  N/A
  1: Strongly disagree
  2: Disagree
  3: Neither agree nor disagree
  4: Agree
  5: Strongly agree
18. Transgender individuals endanger the institution of the family. (N/A=Prefer not to answer)
  N/A
  1: Strongly disagree
  2: Disagree
  3: Neither agree nor disagree
  4: Agree
  5: Strongly agree
19. Transgender individuals should be accepted completely into our society. (N/A=Prefer not to answer)
  N/A
  1: Strongly disagree
  2: Disagree
  3: Neither agree nor disagree
  4: Agree
  5: Strongly agree
20. Transgender individuals should be barred from the teaching profession. (N/A=Prefer not to answer)
  N/A
  1: Strongly disagree
  2: Disagree
  3: Neither agree nor disagree
  4: Agree
  5: Strongly agree
21. There should be no restriction of rights for transgender individuals. (N/A=Prefer not to answer)
  N/A
  1: Strongly disagree
  2: Disagree
  3: Neither agree nor disagree
  4: Agree
  5: Strongly agree
22. I avoid transgender individuals whenever possible. (N/A=Prefer not to answer)
  N/A
  1: Strongly disagree
  2: Disagree
  3: Neither agree nor disagree
  4: Agree
  5: Strongly agree
23. I would feel comfortable working closely with a transgender individual. (N/A=Prefer not to answer)
  N/A
  1: Strongly disagree
  2: Disagree
  3: Neither agree nor disagree
  4: Agree
  5: Strongly agree
24. I would feel comfortable if I learned that my neighbor was a transgender individual. (N/A=Prefer not to answer)
  N/A
  1: Strongly disagree
  2: Disagree
  3: Neither agree nor disagree
  4: Agree
  5: Strongly agree
25. Transgender individuals should be allowed to dress to reflect their gender identity in public. (N/A=Prefer not to answer)
  N/A
  1: Strongly disagree
  2: Disagree
  3: Neither agree nor disagree
  4: Agree
  5: Strongly agree
26. I would feel comfortable if I learned that my best friend was a transgender individual. (N/A=Prefer not to answer)
  N/A
  1: Strongly disagree
  2: Disagree
  3: Neither agree nor disagree
  4: Agree
  5: Strongly agree
27. I would feel uncomfortable if a close family member became romantically involved with a transgender individual. (N/A=Prefer not to answer)
  N/A
  1: Strongly disagree
  2: Disagree
  3: Neither agree nor disagree
  4: Agree
  5: Strongly agree
28. I would feel comfortable if a close family member was a transgender individual. (N/A=Prefer not to answer)
  N/A
  1: Strongly disagree
  2: Disagree
  3: Neither agree nor disagree
  4: Agree
  5: Strongly agree

### Demographics

Instructions: This final section includes demographic questions. It is not a test, so there is no right or wrong answer.

29. What is your gender [choose all that apply]?
  1: Prefer not to answer
  2: Male
  3: Female
  4: Trans man
  5: Trans woman
  6: Nonbinary/Genderqueer
  7: Other
30. Which of the following best describes your hometown?
  1: Prefer not to answer
  2: Large metro (city of 1 million population or more)
  3: Mid-sized metro (city of 250,000–1 million population)
  4: Small-sized metro (city of fewer than 250,000 population)
  5: Urban (20,000–250,000 in the county)
  6: Small urban (i.e., town) (2500–20,000 in the county)
  7: Rural (less than 2500 in county)
31. Which of the following best describes you?
  1: Prefer not to answer
  2: PGY-1
  3: PGY-2
  4: PGY-3
  5: PGY-4 or higher
32. What is your ethnicity?
  1: Prefer not to answer
  2: Hispanic or Latino
  3: Not Hispanic or Latino
33. What is your race? Mark one or more races to indicate what you consider yourself to be.
  1: Prefer not to answer
  2: American Indian or Alaska Native
  3: Asian
  4: Black or African American
  5: Native Hawaiian or Other Pacific Islander
  6: White
  7: Other (If you check this box, please complete question 34)
34. If you checked “Other” for the previous question, please specify your race in this text box. Otherwise, please skip to the next question.
35. How could WVU Medicine improve the care we provide to transgender patients?
36. Do you have any other comments or clarifications you would like to make about this survey?
